# Initial yield of hydrated electron production from water radiolysis based on first-principles calculation

**DOI:** 10.1039/d2ra07274b

**Published:** 2023-03-01

**Authors:** Takeshi Kai, Tomohiro Toigawa, Yusuke Matsuya, Yuho Hirata, Tomoya Tezuka, Hidetsugu Tsuchida, Akinari Yokoya

**Affiliations:** a Nuclear Science and Engineering Center, Japan Atomic Energy Agency 2-4 Shirane Shirakata, Tokai-mura, Naka-gun Ibaraki 319-1195 Japan; b Faculty of Health Sciences, Hokkaido University Kita-12 Nishi-5, Kita-ku Sapporo Hokkaido 060-0812 Japan; c Department of Nuclear Engineering, Kyoto University Nishikyo-ku Kyoto 615-8530 Japan; d Quantum Science and Engineering Center, Kyoto University Gokasho, Uji Kyoto 611-0011 Japan; e Institute for Quantum Life Science, National Institutes for Quantum Science and Technology 2-4 Shirane Shirakata, Tokai-mura, Naka-gun Ibaraki 319-1195 Japan

## Abstract

Many scientific insights into water radiolysis have been applied for developing life science, including radiation-induced phenomena, such as DNA damage and mutation induction or carcinogenesis. However, the generation mechanism of free radicals due to radiolysis remains to be fully understood. Consequently, we have encountered a crucial problem in that the initial yields connecting radiation physics to chemistry must be parameterized. We have been challenged in the development of a simulation tool that can unravel the initial free radical yields, from physical interaction by radiation. The presented code enables the first-principles calculation of low energy secondary electrons resulting from the ionization, in which the secondary electron dynamics are simulated while considering dominant collision and polarization effects in water. In this study, using this code, we predicted the yield ratio between ionization and electronic excitation from a delocalization distribution of secondary electrons. The simulation result presented a theoretical initial yield of hydrated electrons. In radiation physics, the initial yield predicted from parameter analysis of radiolysis experiments in radiation chemistry was successfully reproduced. Our simulation code helps realize a reasonable spatiotemporal connection from radiation physics to chemistry, which would contribute to providing new scientific insights for precise understanding of underlying mechanisms of DNA damage induction.

## Abstract 2

For water radiolysis, conventional simulation methods estimate free radical yields based on the cross-sections. Our results indicated that electron dynamic motion must be further solved to predict the initial yields of hydrated electrons.

## Graphical abstract

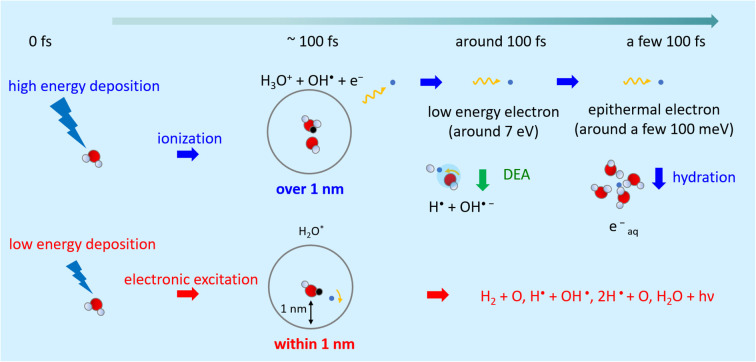

## Introduction

As most biological systems, including the human body, mainly comprise liquid water, a fundamental investigation of the interaction of ionizing radiation with water is crucial for the in-depth understanding of the earliest stages of biological effects, such as DNA damage in genomes,^1^ which are intrinsically related to cell death and mutation induction.^2,3^ As a mechanistic investigation for the earliest stages, DNA damage estimation is a research topic that has garnered worldwide interest. DNA damage yields can be predicted by both physical interactions between radiation and DNA molecules (direct effects) and chemical reactions between DNA molecules and free radicals resulting from water radiolysis (indirect effects). To date, modelling efforts for the scenarios from atomic interaction to early DNA damage induction have been made.^4–6^ However, fundamental scientific insights for the initial production of the radicals are lacking because it is difficult to measure fast phenomena in the femtosecond (fs) order.

When water is irradiated with ionizing radiation, a large number of free radicals are generated nonhomogeneously along the radiation tracks. Generally, secondary electrons are produced by ionization (H_2_O˙^+^ + e^−^), and proton transfer is subsequently caused within 100 fs (ref. 7) (H_3_O^+^ + OH˙ + e^−^). The secondary electrons spread over a region of a few nm. As a result, electrons become delocalized around these parent cations. Hydrated electrons (e_aq_^−^) are formed in these delocalized distributions. After a few 100 fs, hydration of the secondary electrons progresses (H_3_O^+^ + OH˙ + e_aq_^−^).^8–11^ In addition, by the induction of electronic excitation, water molecules dissociate in mainly three types: (OH˙ + H˙), (O + H_2_), and (O + 2H˙).^12^ Oxidative damage to DNA is predicted from these findings of ionization and electronic excitation, however, reductive damage to DNA is still poorly understood.^13^ Since 2000, dissociative electron attachment (DEA) attracted attention as a new DNA damage process induced by low energy electrons.^14^ The induction produces negative dissociation products, such as (O˙^−^ + H_2_), (OH^−^ + H˙) and (H^−^ + OH˙) in gaseous water.^15^ Recent studies found that the DEA is rarely induced in an aqueous solution.^13^ In this regard, the frequency of the DEA in aqueous solution seems to be different from that of the gaseous phase. Even nowadays, the reason for this remains unclear. The DEA is one of the reductive damages. Although this knowledge is still insufficient, its quantitative evaluation is much desired.

The yields of those radicals induced by ionization, electronic excitation, and DEA can be measured using various experimental techniques. Specifically, many experimental results of e_aq_^−^ after a picosecond (ps) order through pulse radiolysis measurements have been reported.^16–23^ In terms of radiation chemistry, the initial yield (*G* value) of e_aq_^−^ at 1 ps was predicted to be 4.15,^17^ 4.4,^18,19^ 4.9 (ref. 23) (1/100 eV), and the latest *G* value is evaluated to be 4.6 (ref. 20 and 21) (1/100 eV). These values were predicted from the experimental results after ps order with solving the Debye–Smoluchowski equation based on the diffusion theory considering coulombic interaction between the radicals.^12,16,23^

In general, the production of these free radicals can be simulated based on the track-structure Monte-Carlo code,^1,24–33^ such as KURBUC,^1,24,25^ TRACEL,^26^ RITRACKS,^27^ PARTRAC,^28^ Geant4-DNA,^29^ and PHITS.^30,31^ The track-structure Monte-Carlo code enables simulating ionizations, electronic excitations, and DEAs created by a primary electron and secondary electrons in liquid water. Here, the ionization, electronic excitation, and DEA coordinates are determined based on the Monte Carlo technique, and the type of free radicals, excluding the e_aq_^−^, can be determined because the general track-structure Monte-Carlo code (such as Geant4-DNA^29^) requires a cutoff energy, which is typically set to be about 7–10 eV. Thereafter, some empirical models based on experimental results for photoionization^33,34^ are generally used to obtain an ultimate delocalization distribution of secondary electrons. Consequently, the delocalization mechanism of the secondary electrons remains unexplained. A reasonable spatiotemporal connection from the physical process to a chemical process related to the generation of e_aq_^−^ has not been realized.

In terms of radiation physics, we have developed a dynamic Monte Carlo code for physical process (hereinafter called dmcc_phys) to analyse the delocalization mechanism of secondary electrons and the generation mechanism of the e_aq_^−^ in liquid water.^35–40^ The secondary electrons not only induce additional ionization and electronic excitation, but also become chemically active species as e_aq_^−^. Our code was developed by implementing a well-known molecular dynamics method in the track-structure Monte-Carlo code. Therefore, the motion of secondary electrons moving in the long-range coulombic field of the parent ions with the electron-water collision is solved by molecular dynamics method based on a Newtonian equation and a Monte-Carlo method based on probability theory of collision (see, Simulation method). This coulombic field is modified by the polarization effect. Therefore, this code includes the polarization effect in Newtonian equation (see, Simulation method). If we eliminate the molecular dynamics method from our code, our code is equal to the track-structure Monte-Carlo code. The electron track structure mode of PHITS,^30,31^ which corresponds to the track-structure Monte-Carlo code, was developed by eliminating the molecular dynamics method based on our code. Hence, our code achieves advanced first-principles calculations for the secondary electrons rather than the track-structure Monte-Carlo code. In the first-principles calculations, the six-dimensional degrees of freedom (position vector (*x*, *y*, *z*) and velocity vector (*v*_*x*_, *v*_*y*_, *v*_*z*_)) of the secondary electron vary from time to time along the molecular dynamics and Monte-Carlo methods. The code includes in collision and polarization effects originating from the molecular polarity of water to demonstrate the first-principles calculations for the secondary electrons. Here, the collision effect is the scattering and energy deposition of the electrons, and the polarization effect is the shielding of the electric field created by the cations produced in the water. It is thought that the collision effect of the secondary electrons moving in coulombic fields created by the cations correlates significantly with the polarization effect wherein some water molecules surround the secondary electrons or cations. This concerted correlation between the collision and polarization effects plays an important role in the delocalization of secondary electrons in water. Hence, we can expect to realize a simulation prediction of the *G* value of e_aq_^−^.

This study aims to explore the generation mechanism of the e_aq_^−^ resulting from the water radiolysis by electron irradiation using a dynamic Monte Carlo code for the physical process. First, to verify our code, we calculated two types of ranges with different definitions in incident electron energies from 0.1 eV to 1 MeV and then compared the corresponding experimental and calculated results of previous studies. Second, we calculated yields with time evolution for ionization + electronic excitation and the DEA induced by an electron injection with 10 keV into water. Third, we explored the unknown concerted correlation in the order of fs from our results for delocalization, energy, and collision frequency distributions of the secondary electrons. Finally, throughout this paper, we discuss the prediction of the initial *G* value of e_aq_^−^ from our calculated results.

### Simulation methods

To well-understand our calculated results, we briefly describe some differences between the track-structure Monte-Carlo code^1,24–33^ and our codes. The track-structure Monte-Carlo code is capable of locating various free radicals on the electron track, but utilizes some model^33,34^ in locating e_aq_^−^. Although our code has difficulty locating all free radicals, it is capable of identifying the ionization process (H_3_O^+^ + OH˙ + e^−^). The track-structure Monte-Carlo code adopts the kinetic method, whereas our code adopts the dynamic method. That is, the electron motion in the dynamical coulombic fields that change from moment to moment in water can be analysed. Therefore, we can demonstrate a concerted correlation between the collision and polarization effects. These track-structure Monte-Carlo codes must set generally the cutoff energy to stop the electron transport. Our code solves the relativistic Newtonian equation and outputs the spatial and energy distributions of the primary and secondary electrons at each time.^40^ Therefore, the cutoff time should be set, but the setting of the cutoff energy is unnecessary.

To demonstrate the novelty of our method and results, we briefly describe the history of code development and improvements. We reported dmcc_phys in 2014.^35^ First, we used the cross-sections in gaseous phases.^35^ In 2015, we evaluated the intramolecular vibration excitation cross-section of liquid water and also calculated the intermolecular vibration and rotation excitation cross-sections of liquid water.^36^ We also calculated the ionization and electronic excitation cross-section of liquid water. For simulations of water radiolysis,^40^ we assumed that secondary electrons are ejected from water molecules if ionization (1b_1_, 3a_1_, 1b_2_, 2a_1_, and 1a_1_ ionizations) are induced. The initial energy of the secondary electron was sampled from the single-differential cross-sections of the gas phase.^40^

In this study, we improved a model of potential energy created by electrons or cations. We also evaluated the dielectric response of water by Fourier transform of a complex dielectric function. We provided energy to the electrons by induction of ionization and electronic excitations (A^1^B_1_, B^1^A_1_, Rydberg (A + B), Rydberg (C + D), diffuse band, and collective excitations). Here the ionized and excited electrons are collectively defined as the secondary electrons. To determine the initial energy of the secondary electrons, the deposition energy is sampled from the energy loss function.

### Fundamental physics

**Fig. 1:**
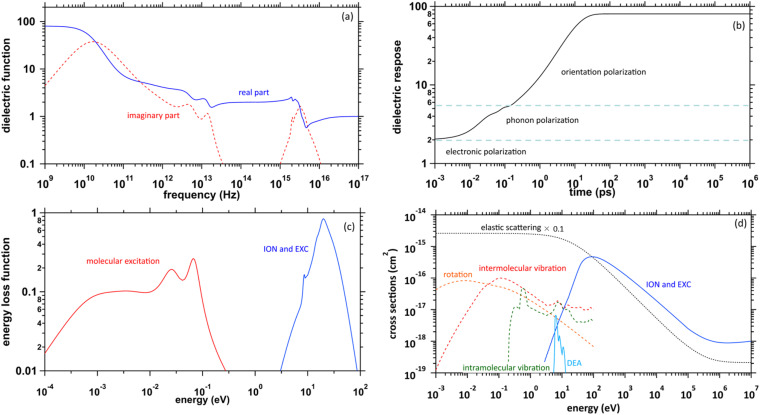
(a) Previously reported complex dielectric function of water. (b) Dielectric response of water calculated in our study. (c) Energy loss function of water. (d) Elastic and inelastic scattering cross-sections by electron impacts implemented into dmcc_phys of water.

The physicochemical properties of water, which become the core elements of this study, are given by the complex dielectric function, which can be obtained from measurements of optical frequency over a wide range.^41,42^Fig. 1(a) presents the previous results of the complex dielectric function. The fitting parameters of the complex dielectric function for ionization and electronic excitations have been reported.^26^ For molecular excitations, the fitting parameters for intermolecular vibration and rotation excitations have also been reported.^42^ The dielectric response of water, as shown in Fig. 1(b), was given by the Fourier transform of the complex dielectric function. The result corresponds to the time evolution of the relative dielectric constant of water. This dielectric response begins to increase moderately from a few fs due to phonon polarization and then increases from a few 100 fs due to orientation polarization and is completed in a few 10 ps. The timescale corresponds to that of hydration dynamics wherein the secondary electrons are converted into e_aq_^−^. Further, the complex dielectric function can be converted into an energy loss function corresponding to the energy absorption efficiency (Fig. 1(c)). This allowed the calculation of electron impact cross-sections.^43^

### Collision algorithm for electrons

Fig. 1(d) presents the electron impact cross-sections used in this study. Ionization, electronic excitation, and DEA cross-sections are involved in locating free radicals, while intramolecular vibration, intermolecular vibration, rotation excitations cross-sections, and elastic scattering cross-section are involved in locating e_aq_^−^. The ionization and electronic excitation cross-sections of liquid water below 100 keV were calculated using the energy loss function (*i.e.*, ION and EXC) of Fig. 1(c). The figure also shows the total cross-section obtained by summing the 11 processes. It is well known that the cross sections of the gas and liquid phases water for ionization and electronic excitation are different.^44^ In high energy region, however, the total ionization and electronic excitation cross-section of gas phase is equal to that of liquid phase by the sum rule of oscillator strength.^26,32^ Although the total cross-section of the gas phase water above 100 keV was used,^35^ we connected each cross-section of the 11 processes while maintaining the ratio of each process of the liquid phase. Only a negligible DEA frequency in an aqueous solution^13^ has been reported. In our previous study combining microdroplet and mass spectrometry,^45^ we found that when an aqueous solution simulating a living system is irradiated with carbon ions, while many cations are produced, a few anions are also produced. The DEA is one of the possible mechanisms for the formation of these anions. However, DEA frequencies will differ significantly between liquid and gas phases. Thus, we assumed that the DEA cross-section in the liquid phase was to be reduced to 1/20 of that in the gas phase shown in the Fig. 1(d). Consequently, uncertainties are included in the current situation regarding the DEA results. Now, we analyse unpublished experimental results measured by liquid water jet and mass spectrometry to eliminate this uncertainty. It is expected that our future developments will resolve this uncertainty. The intramolecular vibration excitation cross-sections were evaluated by combining the data of the gas phase and those of amorphous ice.^36^ The intermolecular vibration and rotational excitation cross-sections were calculated using the energy loss function (molecular excitation) shown in Fig. 1(c).^36^ The elastic scattering cross-section *σ*_elas_ reported by Moliere^46^ was used.

When elastic scattering is induced, there is no energy change for the relative motions of an electron and a water molecule, but the energy for the motion of center-of-mass system changes.^47,48^ This phenomenon becomes effective in an extremely low energy region and is evaluated by the momentum transition cross-section *σ*_mom_, which can be obtained from the differential cross-section *q*(*θ*) of elastic scattering.^47,48^1
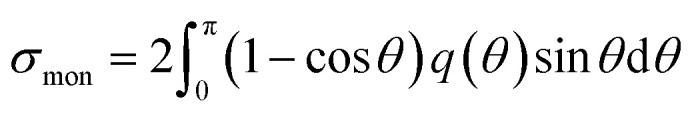
here, *θ* is the scattering angle. Using *σ*_mom_ of eqn (1), the energy transfer is given as follows:^40,47,48^2
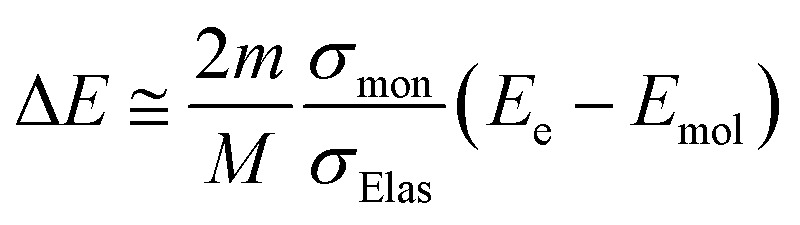
where *m* and *M* are the mass of the electrons and water molecules, respectively; *E*_e_ and *E*_mol_ are the kinetic energies of the electrons and water molecules, respectively; and *E*_mol_ is sampled from the Maxwell distribution of 300 K. Therefore, in the case of *E*_e_ > *E*_mol_, the secondary electrons provide energy to water, whereas in the case of *E*_e_ < *E*_mol_, the secondary electrons receive energy from water. Although Δ*E* is approximately μeV, the secondary electrons become thermalized by many collisions. The track-structure Monte-Carlo codes provide one step distance of the electrons that move in water as Δ*s* = −*λ* ln(*k*).^26^ Here, *λ* is a mean free path that is obtained from the total cross-section *σ* and atomic density *N*_atom_, such as *λ* = 1/*σN*_atom_; and *k* is a uniform random number. In our code, we assumed that the collision between an electron and water is induced when the following conditions of eqn (3) are satisfied:3
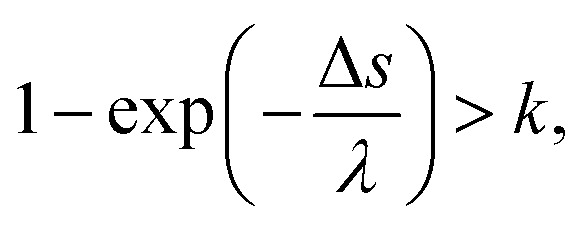
where Δ*s* = *v*Δ*t*, *v* is the absolute value of the velocity, and Δ*t* is a time step that is set to 1 as. After the collision position is known, the collision process is determined by sampling the ratio of the cross-sections shown in Fig. 1(d). In this way, we developed a time-dependent collision algorithm.

### Dynamic algorithm of electrons

**Fig. 2:**
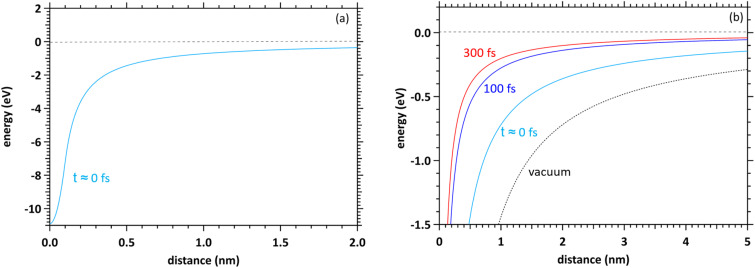
(a) Potential energy included in the polarization effect of the finite-size particle model assumed in this code immediately after energy deposition. (b) Time evolution of potential energy included in the polarization effect of the finite-size particle model assumed in this code.

Initially, we describe the case where ionization and electronic excitation are induced. We assumed that the electrons and cations are finite-size particles with radius a having negative and positive charges *e* (finite-size particle model).^40^ The particle radius was set to 0.99 nm to reproduce the ionization energy of 10.9 eV,^49^ and the position of a minimum of the potential energy (−10.9 eV) is allocated at the position where ionization or electronic excitation is induced as shown in Fig. 2(a). When the potential of the cation is expressed in spherical coordinates, it can be presented in eqn (4).4
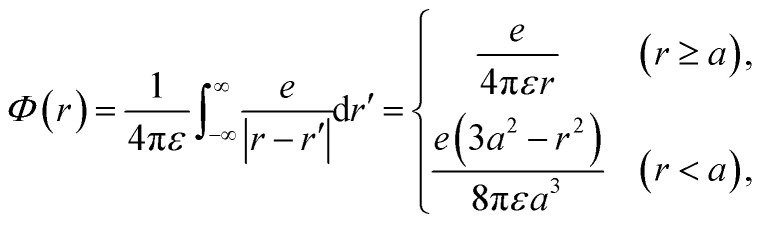
here, *e* is an element charge, *ε* is *ε*_0_ × *ε*_r_(*t*), *ε*_0_ is the dielectric constant of the vacuum, and *ε*_r_(*t*) is the time evolution of the relative dielectric constant of water, which is given by the dielectric response in Fig. 1(b). By the polarization effect, the potential energy changes over time (Fig. 2(b)). The secondary electron is allocated at the position where ionization or electronic excitation is induced, and a velocity vector is given. The velocity vectors of the primary and secondary electrons are obtained from the relationship between momentum and energy conservation.^26^ Here, to obtain the absolute value of the velocity of the secondary electron, we sample the deposition energy of the secondary electrons from the energy loss function presented in Fig. 1(c). When the DEA is induced, an anion is formed at this position, and the anion is fixed at this position. When the elastic scattering is induced, we sample the scattering angle from the differential cross-section *q*(*θ*). When the molecular excitations were induced, we assumed that the scattering angle did not change. The dynamic motion with the collision of the primary and secondary electrons can be obtained by solving the relativistic Newtonian eqn (5).5
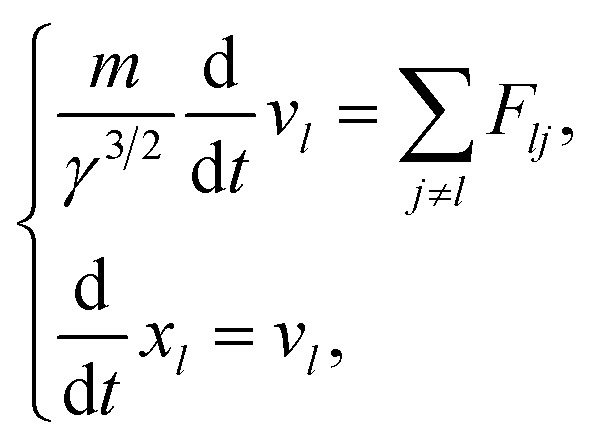
wherein
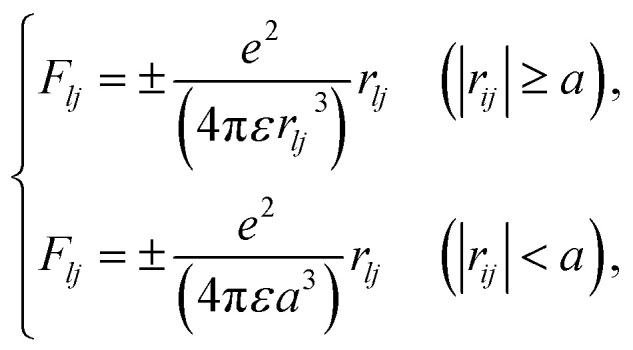
here, *γ* is (1 − *v*^2^/*c*^2^), where *c* is the speed of light. Here, we assume that radicals do not move. We considered three-dimensional coulombic interactions between pairs of the parent ions and the secondary electrons.

As our code comprises the collision and dynamic algorithms, the motion of the electrons moving in the dynamic coulombic field created by the parent ion while colliding with water can be calculated. The coulombic field is shielded along the dielectric response (Fig. 1(b)), and we simulate hydration dynamics by the shielding. Although proton transfer occurs within 100 fs (ref. 7) (H_3_O^+^ + OH˙ + e^−^), the cations are fixed in the generation position. This is because the Born–Oppenheimer approximation^50^ allows electrons to follow the motion of protons. The number of calculation trials was adapted to reach a statistical uncertainty of much less than 1%.

### Flowchart

**Fig. 3:**
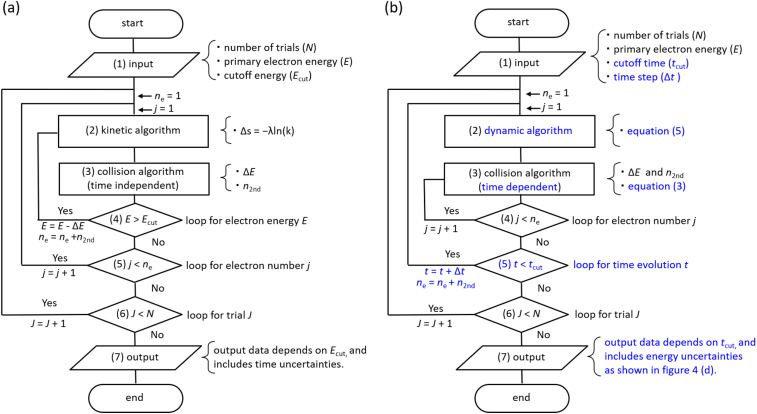
(a) Flowchart of typical track-structure Monte-Carlo codes.^1,24–33^ (b) Flowchart of dmcc_phys.

Fig. 3(a) shows a flowchart of typical track-structure Monte-Carlo codes,^1,24–33^ where the inputs to the track-structure Monte-Carlo code are the number of trials (*N*), primary electron energy (*E*), and electron cutoff energy (*E*_cut_). (2) In the kinetic algorithm, electrons are transported by Δ*s* = −*λ* ln (*k*),^26^ where Δ*s* is the one step distance, *λ* is the mean free path, and *k* is a uniform random number. (3) In the collision algorithm, the energy loss Δ*E* of the electrons and the number of generated electrons *n*_2nd_ are obtained. Processes (2) and (3) are repeated until the primary electron energy reaches the cutoff energy. Then, for the secondary electrons generated, repeat steps (2) and (3) in the same manner as for the primary electrons until the electron energy reaches the cutoff energy. This calculation for processes (2)–(5) is repeated for all secondary electrons. Once these calculations are complete, move on to the next trial *J* = *J* + 1 (process (6)). All calculations are completed when the statistical uncertainties in the results are sufficiently small. In the track-structure Monte-Carlo code, the calculated result depends on the cutoff energy. Therefore, the calculated results include time uncertainties.

The above calculations identify the location where ionization or electronic excitation is induced, and this information is used to identify the type of the free radicals produced. However, it is difficult to identify the location of the e_aq_^−^. Therefore, some models^33,34^ ware generally used when solving electron delocalization distribution for physicochemical processes. Here the positions of all radicals are obtained and the subsequent diffusion and reactions of the radicals are calculated by chemical code.^12,16,22^

Fig. 3(b) shows the flowchart of our code, where the inputs to our code are the number of trials (*N*), the primary electron energy (*E*), the cutoff time (*t*_cut_) of the calculation, and the time step Δ*t* (1 attosecond). (2) In the dynamic algorithm, the dynamic behaviour of the primary and secondary electrons is simultaneously solved for each time step Δ*t* according to eqn (5). (3) In the collision algorithm, electron water collisions are determined by eqn (3), and if a collision occurs, the electron energy loss Δ*E* and the number of generated electrons *n*_2nd_ are obtained. The process (3) is repeated for the number of *n*_2nd_. After the process (3) is completed, we move on to the next time *t* = *t* + Δ*t*. This process (2)–(5) is repeated until the cutoff time is reached. Once these calculations are complete, move on to the next trial *J* = *J* + 1 (process (6)). All calculations are completed when the statistical uncertainty of the results is sufficiently small. In our code, the calculated result depends on the cutoff time. Therefore, the calculated results include energy uncertainties.

Our code calculates electron deceleration, thermalization, delocalization, and initial hydration using first principles calculations. This identifies the location of e_aq_^−^, and we can challenge to evaluate the initial yield of hydrated electrons. Treatment of the free radicals other than e_aq_^−^ and calculations for diffusion and reactions of the free radicals are the subject of future work.

As we have mentioned, water radiolysis is simulated in three stages: physical, physicochemical, and chemical processes. The physical process is the energy deposition into water by radiation, and calculates the position and yield of ionization and electronic excitation induced. Here, track-structure Monte-Carlo codes such as Geant4-DNA^29^ are used, and the flowchart is shown in Fig. 3(a). The cutoff energies of Geant4-DNA^29^ and a code reported by Pimblott *et al.*^33^ are 1–10 eV, and 5–25 eV, respectively. The physicochemical processes locate the various radicals based on ionization and electronic excitation information. For the position of e_aq_^−^, the models reported by Ritchie *et al.*,^34^ and Pimblott and LaVerne^33^ is generally used because track-structure Monte-Carlo codes do not calculate sufficient slowing down processes of secondary electrons. For chemical processes that calculate radical diffusion and reactions, independent reaction time, random reaction time, and step-by-step methods have been developed.^12,16,22^ Our dmcc_phys simulates the physical as well as physicochemical processes according to the flowchart shown in Fig. 3(b). When simulating an electron of 10 keV, the track-structure Monte-Carlo code completes the calculation in 15 seconds, while our code takes 8 minutes. The differences from typical track-structure Monte-Carlo codes are indicated in blue. The dmcc_phys does not set cutoff energy. The energy distribution of secondary electrons is set to asymptote to the Maxwellian of 300 K over time by the eqn (2). Therefore, it is necessary to set the cutoff time, which is set to 300 fs. There are also codes^51^ that use cutoff time, such as our code. While previous works^12,16^ deal with a variety of decomposition processes, the process treated in detail in this study is so far limited to ionization process (H_3_O^+^ + OH˙ + e^−^). Our dmcc_phys cannot simulate chemical processes, and therefore cannot be compared to chemical codes.^12,16,22^ We are currently developing an original chemical code (dynamic Monte Carlo code for chemical process or hereinafter called dmcc_chem), which is based on the step-by-step method.

## Results

To verify our code, we present the calculated results of the ranges of a primary electron (Subsection, Primary electron), as well as the secondary electrons dominated by physicochemical properties of water, such as the collision and polarization effects (Subsection, Secondary electrons).

### Primary electron

**Fig. 4:**
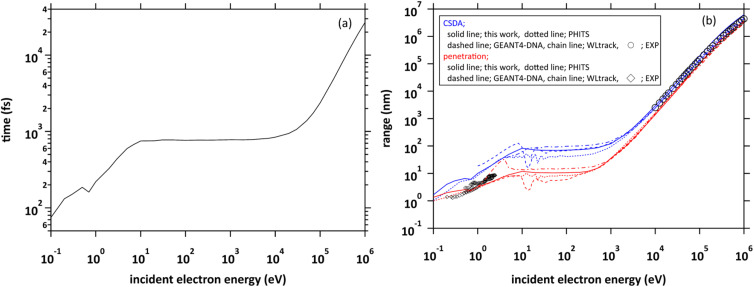
(a) Thermalization times of incident electrons in water in the energy region from 0.1 eV to 1 MeV. (b) Two types of range of incident electrons in water in the energy region from 0.1 eV to 1 MeV.

We projected a primary electron from the origin along the *z*-axis. In the calculation of the range, we first investigated the cutoff time in which the electron decelerates to 25.6 meV (300 K). We show the cutoff time of the primary electrons in the incident energy from 0.1 eV to 1 MeV in Fig. 4(a). Generally, the ejection energies of the secondary electrons are a few 10 eV. We found in the figure that the thermalization time of the electrons with an energy of a few 10 eV is less than 800 fs. The typical generation time of e_aq_^−^ is considered to be a few 100 fs.^8–11^ These facts suggest that secondary electrons generated in water radiolysis begin to transition to e_aq_^−^ before they reach full thermal equilibrium. Therefore, when analysing 10 keV electron, we must set the cutoff time as 300 fs because the secondary electrons begin to hydrate from around this time.^8–11^ When the electron energy exceeds a few 10 keV, the thermalization time tends to increase rapidly.

Fig. 4(b) shows the calculated results of the ranges. Continuous slowing down approximation (CSDA) is defined as the sum of the distances between the positions of inelastic scattering induced along the primary electron track. Meanwhile, penetration is defined as a straight-line distance between the starting and ending points of the primary electron. Therefore, CSDA depends on the accuracy of the stopping powers and collision cross sections, while penetration depends on the accuracy of the scattering angle as well as the stopping powers and collision cross section. In the incident energies above 1 keV, the ranges calculated by dmcc_phys were in good agreement with the previous experimental and calculation results.^51–55^ In the energy below 1 keV, there are differences among these calculation results, but the trend is similar. The differences depend largely on the molecular excitation cross-sections and the setting of the cutoff energy. Our simulation results also showed a good agreement with the experimental results^56^ in an extremely low energy region around 1 eV. In this way, we verified the fundamental features of our code through intercomparison among our dmcc_phys code, other simulations, and the corresponding experimental data.

### Secondary electrons

**Fig. 5:**
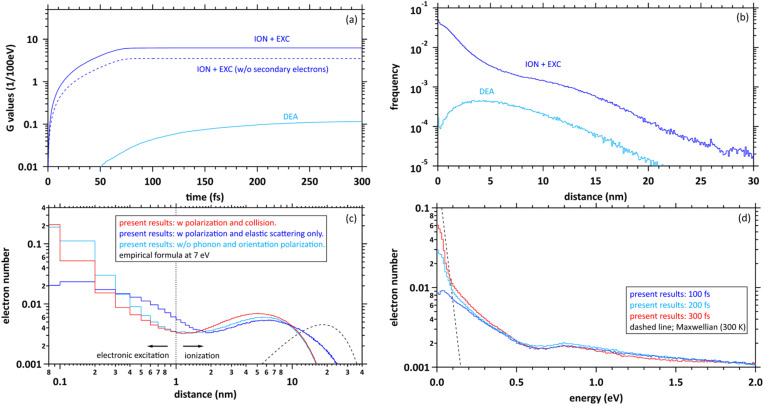
(a) *G* values with time evolution of ionization (ION) + electronic excitation (EXC) and dissociative electron attachment (DEA), (b) collision frequency distribution of ION + EXC, and DEA during 300 fs, (c) delocalization distributions of the secondary electrons at 300 fs. The horizontal axis shows the distance from the ionic core to secondary electrons. (d) Energy distributions of secondary electrons at 100, 200, and 300 fs. In these figures, the distribution results are shown as spherical coordinates with a spatial mesh Δ*r* = 0.1 nm and an energy mesh Δ*E* = 10 meV. All-solid angle meshes Δ*Ω* in the Δ*r* are integrated.

Here, to explore the delocalization mechanism of the secondary electrons and generation mechanism of e_aq_^−^, we irradiated water with 10 keV electron. We analysed spatiotemporally the dynamic motion of the secondary electrons in water. We set the cutoff time as 300 fs. Fig. 5(a) shows the *G* values with time evolution of ionization + electronic excitation (ION + EXC) and the DEA. The ionization and electronic excitation cannot be induced after approximately 100 fs, resulting in a *G* value of 6.24. If the secondary electron production is ignored, the *G* value becomes lower (*i.e.*, 3.51). Therefore, the contribution to the *G* value of the secondary electrons is 43.8%. Meanwhile, the DEA was induced from about 50 fs, and the *G* value gradually converged to 0.115 at 300 fs. Once the ratio of electronic excitation to ionization is known, these values may be used to predict the time-evolution yield of radicals on the femtosecond order.

Fig. 5(b) shows the collision frequency distribution wherein the secondary electrons induce additional ionization, electronic excitation, and DEA during 300 fs. The horizontal axis indicates the distance from the parent ionic core. When the deposition energy exceed 20 eV, additional ionization and electronic excitation are induced by the secondary electrons. The deposition energy was sampling from the energy loss function shown in the Fig. 1(c). For the results of electronic excitation and ionization, the yields within 6 nm of the parent ion are mainly contributed by outer-shell ionization, while the yields above 6 nm are mainly contributed by about 500 eV Auger electrons generated by inner-shell ionization. The collision frequency of DEA is spatially spread because the process requires some deceleration of the generated secondary electrons. The DEA is rarely induced when the electron energy is about 7 eV. The ionization and electronic excitation are likely to be induced near the parent cations where the secondary electrons are generated, while the DEA is hardly induced near the parent cations. The rate induced within 1 nm of the cations was 36.8%, whereas that induced over 1 nm was 63.2%. These features provide us with a fundamental scientific insight for analysing the sites of DNA damage.^40^

Fig. 5(c) shows the calculated results of the delocalization distributions of the secondary electrons at 300 fs. The horizontal axis indicates the distance from the parent ionic core. We presented calculated results for time evolution of the delocalization distributions of secondary electrons at 1 keV electron in our previous paper.^40^ The calculated results did not include 6 processes of A^1^B_1_, B^1^A_1_, Rydberg (A + B), Rydberg (C + D), diffuse band, and collective excitations. In our previous work,^40^ it was found that a part of secondary electrons is recaptured into the parent ions even when the deposition energy exceeds the ionization energy (10.9 eV (ref. 49)) in the order of 100 fs. From the results with polarization and collision (red line), we found that the distributions are roughly divided into two regions. The distribution within a radius of 1 nm shows an exponential distribution, where electrons induced by deposition energy are strongly bound to the parent ion, and the distribution above a radius of 1 nm shows a Gaussian, where the electrons are in diffuse motion in the water. The region within 1 nm of the parent cation is mainly composed of the secondary electrons not only excited to the A^1^B_1_ and B^1^A_1_ states (excitation energy, 8.4 eV (ref. 26) and 10.1 eV (ref. 26)) of water molecules but also the electron recapture.^40^ Thus, the electron recapture within a few 100 fs plays an important role in determining the ratio between ionization and electronic excitation. The electron recapture will strongly depend on the polarization and collision effects. In the region over 1 nm, the delocalization distribution shows a maximum value of approximately 6 nm. Using the empirical formula,^34^ the maximum value is shown at about 20 nm when the energy of the electron is 7 eV (black line). From these results without phonon and orientation polarizations, the distribution strongly shifts to parent cations due to neglect of shielding of the coulombic forces created by the cations (light blue line). From these results with polarization and elastic scattering only, the secondary electrons are holistically distributed outward due to the neglect of the deceleration of the secondary electrons (blue line). From the comparison with and without the polarization and collision effects, these simulation results indicated that the concerted correlation between the polarization and collision effects plays an important role in the delocalization of secondary electrons.

Fig. 5(d) shows the calculated results of the energy distributions of the secondary electrons at 100, 200, and 300 fs. The horizontal axis indicates the kinetic energy of secondary elections. Our code is time-dependent, resulting in energy uncertainties at each time as shown in the figure. On the other hand, since the track-structure Monte-Carlo code is energy-dependent, the time uncertainties appear when the energy is determined. The energy components below 0.1 eV approach Maxwellian of 300 K after a few 100 fs, indicating the energy distribution of electrons in diffuse motion in water, while those above 0.1 eV indicate the energy distribution of electrons strongly bound to the parent ion. From the results in Fig. 4(a), the secondary electrons become thermalized at less than 800 fs, while the secondary electrons are gradually converted to e_aq_^−^ in a few 100 fs.^8–12^ From this evidence, we suggest that the secondary electrons become epithermal electrons after a few 100 fs and gradually convert to e_aq_^−^ without *via* thermal electrons.

After a few 100 fs or higher, an orientation polarization becomes dominant, hydration proceeds rapidly, and the dielectric response is completed in a few 10 ps as shown in the Fig. 1(b). The chemical reaction of the e_aq_^−^ proceeds after a few 100 ps.^17^ The concentration of the radicals becomes homogeneous after a few 100 ns, and about 60% of the e_aq_^−^ disappears after the chemical reactions after 1 μs.^17^ We should note that rate of solvated electron disappearance depends on not only time itself but also the concentration of chemical components. For example, the lifetime of the solvated electrons in ultrapure deoxygenated water at very small doses per pulse is long, and can be more than a few tens μs.

## Discussion

We discuss the initial *G* value of the e_aq_^−^. Since the chemical reaction of the e_aq_^−^ proceeds after a few 100 ps,^17^ the position of the e_aq_^−^ hardly changes between a few 100 fs and a few 10 ps during the hydration. Therefore, we can consider that the delocalization distribution of the secondary electrons is almost the same as the initial distribution of the e_aq_^−^. From the calculated results in Fig. 5(a), the *G* value for ionization + electronic excitation is 6.24 at a few 100 fs. Hence, the ratio of the ionization and the electronic excitation can be determined from the delocalization distribution in Fig. 5(c). We assumed that electrons within a radius of 1 nm are determined to be excited because they are strongly bound to the parent ion, while electrons above a radius of 1 nm are determined to be ionized because they are less bound to the parent ion. Based on the assumption, the ratio of the distributions within 1 nm and above 1 nm was 1 : 2. We thought that the ratio corresponds to that of the electronic excitation and ionization. Multiplying this ratio by the *G* value of the ionization + the electronic excitation, the *G* value for the ionization can be deduced to be 4.16. Moreover, the electrons will be reduced by the DEA, of which the *G* value was 0.115 at 300 fs. Thus, the residual corresponds to the *G* value of e_aq_^−^. We found that the initial *G* value of e_aq_^−^ is 4.05 at a few 100 fs. The value is almost the same as 4.15 (ref. 17) although our value is less than experimental results of 4.4,^18,19^ 4.6,^20,21^ or 4.9.^23^ Our value includes two assumptions. Re-evaluating not only the DEA cross section of the liquid phase experimentally but also the radius that distinguishes electronic excitation from ionization in terms of the reaction radius^12^ would improve the results. The typical reaction radius for e_aq_^−^ and H_3_O^+^ was 0.75 nm instead of 1 nm.^12^

Hence, we also successfully verified our code from the viewpoint of the number of the secondary electrons. This verification clarifies both the accuracy of physical data as shown in the Fig. 1 and the validity of a model potential, including the polarization effects as shown in the Fig. 2. The *G* value for the DEA becomes a few percentage of e_aq_^−^. On the femtosecond order, OH˙ radical is produced mainly by the ionization process focused on in this study. Our predicted yield for this process is 4.16, as described above. But the radical is also produced *via* electronic excitation processes. Although many results for branching ratios of the excitation processes have been reported,^12,26,32^ a quantitative evaluation for branching ratios is our future work.

**Fig. 6:**
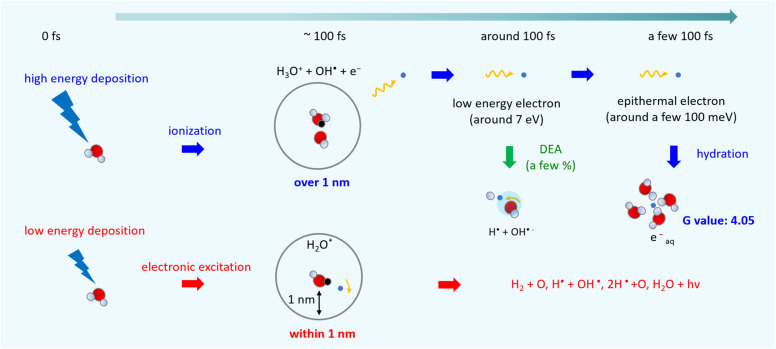
Illustration of the summary of this study.

Fig. 6 shows an illustration of a summary of this study. When low energy around ionization energy 10.9 eV is deposited to water, electronic excitation would be induced, with induced electrons strongly bound to the parent ion. In this study, electronic excitation is determined when electrons are distributed within 1 nm of the parent ion (Fig. 5(c)). In this case, water molecules dissociate into various types of radicals. When high energy above ionization energy 10.9 eV is deposited to water, induced electrons will eject from the parent ion, and ionization will occur. In this study, ionization is determined when the electrons are separated from the parent ion by more than 1 nm (Fig. 5(c)). After the electron ejection, proton transfer occurs within 100 fs, and DEA is induced when the electron energy is about 7 eV at around 100 fs. The *G* value for the DEA (0.115) is a few percentage of the *G* value for ionization + electronic excitation (6.24) under the liquid water (Fig. 5(a)). The secondary electrons become epithermal electrons after a few 100 fs (Fig. 5(d)) and begin to change from the epithermal electrons to e_aq_^−^ at about 6 nm from the parent cations (Fig. 5(c)). From the analysis of the delocalization distribution, the ratio of electronic excitation and ionization was 1 : 2 (Fig. 5(c)) at a few 100 fs, and the *G* value of e_aq_^−^ was predicted to be 4.05.

Conventional track-structure Monte-Carlo simulations estimate the *G* value of each radical based on the cross-sections for the ionization and electronic excitation.^1,24–33^ Our results indicated that the dynamic motion of the secondary electrons must be further solved to predict the *G* value. Our follow-up study also focuses on developing the dmcc_chem in which can simulate the diffusion and reaction of the free radicals. By connecting dmcc_phys and dmcc_chem spatiotemporally, those codes enable realizing a more dense link between radiation physics and chemistry in the future. The link is expected to provide us with a much deeper understanding for unclear radical generation mechanism.

Although our work might contribute to other research fields, such as nuclear energy industry, we have intended to obtain water radiolysis aspects related to radiobiological effects for a decade. We reported many scientific insights especially for the role of secondary electrons in the water radiolysis. These must be an indispensable knowledge for understanding DNA damage formation as a starting point for radiobiological effects in our previous works.^35–40^ In turn, these insights can be applied to radiation chemistry research related to the nuclear fuel cycle.^57^ Radiation fields generated by the wide range of radioactive species contained in spent nuclear fuel are complex one containing low linear-energy-transfer (LET) radiation (β or γ rays) and high LET radiation (α rays).^58^ The high-LET radiation fields are densely ionized, and the dynamics algorithm of our code, which can simulate the effects of coulombic fields, will be essential to analyse the initial radiolytic process. Currently, we are developing the code only for electrons (low-LET radiation), but we believe that developing the code available for high-LET radiations such as α rays is one of the important issues in the future. Such code development for high-LET radiations (α rays and carbon ions) can provide new fundamental insights not only for the nuclear fuel cycle but also for particle therapy. But these should be performed in the future study.

## Conclusions

In this paper, we proposed many scientific insights for secondary electrons. We found that the *G* value for ionization + electronic excitation is 6.24 at a few 100 fs, the electronic excitation and ionization ratio is 1 : 2, and the *G* value for the DEA becomes a few percentage of that for the ionization. From these results, we predicted that the initial *G* value of e_aq_^−^ becomes 4.05 at 300 fs. The result is consistent with 4.15, predicted from the radiation chemistry viewpoint. Our results suggest that the database presented in Fig. 1 should be used to solve the dynamic motion of the secondary electrons using the first-principles calculation with the prediction of the initial *G* value. Our method will become a game-changer in radiation physics and chemistry that can provide much scientific insights for the unclear mechanism of free radical generation. At present, our prediction is limited to the initial *G* value of e_aq_^−^. In the near future, we will develop a code system connected with dmcc_phys and dmcc_chem. The system should be able to predict the initial *G* values of other free radicals. The prediction system is expected to provide a much deeper understanding on estimating radiation DNA damage.

## Author contributions

T. Kai and T. Toigawa designed this work. T. Kai developed the dynamic Monte Carlo code physical process and performed all calculations. Y. Matsuya and Y. Hirata contributed to the discussion for the development of the code and radiation physics. H. Tsuchida and T. Tezuka contributed to the discussion of radiation physics. T. Toigawa contributed to the discussion of radiation chemistry. A. Yokoya supervised this study. T. Kai wrote the manuscript. All authors contributed to the discussion of this study and have reviewed the manuscript.

## Conflicts of interest

There are no conflicts to declare.

## Supplementary Material
